# The Association of Sarcopenia and Visceral Obesity with Lean Nonalcoholic Fatty Liver Disease in Chinese Patients with Type 2 Diabetes Mellitus

**DOI:** 10.1155/2022/2229139

**Published:** 2022-11-04

**Authors:** Xingxing Zhang, Zhiying He, Qiya Si, Xiang Hu, Lijuan Yang, Xiao Gu, Linjia Du, Lei Wang, Linyu Pan, Yingqian Li, Jing Li, Bo Yang, Xuejiang Gu

**Affiliations:** ^1^Department of Endocrinology and Metabolism, The First Affiliated Hospital of Wenzhou Medical University, Wenzhou, 325000 Zhejiang, China; ^2^Institute of Life Sciences & Biomedical Collaborative Innovation Center of Zhejiang Province, Wenzhou University, Wenzhou 325035, China; ^3^School of Public Health and Management, Wenzhou Medical University, Wenzhou, Zhejiang, China

## Abstract

**Background:**

Few studies have specifically observed the relationship of sarcopenia, visceral obesity, or their joint effects with lean NAFLD in patients with diabetes. We aimed to investigate the associations of lean NAFLD with sarcopenia, visceral obesity, and sarcopenic visceral obesity (SV) in Chinese patients with type 2 diabetes mellitus (T2DM).

**Methods:**

Altogether, 1,112 T2DM patients with BMI <25 kg/m^2^ were enrolled, and 33.18% of them were diagnosed with lean NAFLD by abdominal ultrasonography. Body composition markers were measured by bioelectrical impedance (BIA). Skeletal muscle mass index (SMI) was calculated as appendicular skeletal muscle mass (ASM) divided by weight, and sarcopenia was defined as SMI < 1 standard deviation (SD) below the sex-specific average for a young reference population. Visceral obesity was defined as visceral fat area (VFA) ≥ 100 cm^2^. Participants were categorized into one of the four body composition groups: nonsarcopenia/nonvisceral obesity (NN), nonsarcopenia/visceral obesity (NV), sarcopenia/nonvisceral obesity (SN), and SV.

**Results:**

Compared to those in the NN group, patients in the NV and SN groups had a higher risk of lean NAFLD after full adjustments (NV: OR = 1.74; 95% CI: 1.09, 2.78; SN: OR =2.07; 95% CI: 1.23, 3.46). Of note, patients in the SV group had the highest odds of lean NAFLD (OR = 3.29; 95% CI: 2.10, 5.17). There were no significant interaction effects between sarcopenia and metabolic risk factors on prevalent lean NAFLD.

**Conclusions:**

The current study demonstrated that SV was more closely associated with higher prevalent lean NAFLD than sarcopenia or visceral obesity alone in Chinese patients with T2DM. Besides, the harmful effect of sarcopenia on lean NAFLD was not influenced by visceral obesity or other metabolic risk factors. We hypothesize that increasing skeletal muscle mass more than just reducing visceral fat might be more optimal for the prevention and management of lean NAFLD, which needs further investigation in future studies.

## 1. Introduction

Nonalcoholic fatty liver disease (NAFLD), referred as excessive lipid accumulation in the liver, is the most common cause of chronic liver disease, with a high prevalence ranging from 25% to 45% globally [1]. It is characterized by dysfunctional metabolic milieu with increasing risks of end-stage liver disease, cardiovascular diseases, and mortality [2]. Though NAFLD is typically accompanied with obesity, a growing number of patients with NAFLD do not suffer from overweight/obesity, a condition known as “lean NAFLD” or “nonobese NAFLD” [3]. Subjects with lean NAFLD have similar prognoses to those with obese NAFLD and have a worse long-term clinical outcome than patients without NAFLD [4]. However, NAFLD is usually overlooked in lean subjects, which may in turn further increase the harmful effect of lean NAFLD. Besides, a study even showed that lean NAFLD was more strongly associated with a higher risk of diabetes than obese NAFLD [5]. Thus, more efforts are needed to better discover possible risk factors and plausible managements for lean NAFLD.

Sarcopenia, a loss of muscle mass or/and strength, being closely related to insulin resistance (IR), systemic inflammation, and myokine secretion, was proved to be a strong risk factor for lean NAFLD [6, 7]. The concurrence of sarcopenia and obesity (general or visceral), a situation called “sarcopenic obesity (SO),” has been considered to have much more adverse impacts upon physical disability and cardiometabolic disorders than sarcopenia or obesity alone [8, 9]. In addition, sarcopenia and excessive fat accumulation often coexist in patients with NAFLD [10], which may attribute to the complicated interplay between muscle mass, adipose tissue, and liver, known as the muscle-liver-adipose tissue axis [11, 12]. There are several studies based on the general population that showed SO was independently associated with an increased risk of NAFLD [13–15]. However, few studies have specifically observed the association between SO and lean NAFLD [16].

Evidences suggested that the presence of T2DM may have promoted the development of lean NAFLD, leading it to nonalcoholic steatohepatitis (NASH) or, worse, liver cirrhosis [17]. On the other side, it was demonstrated that SO was more prevalent in individuals with T2DM compared to those without T2DM [18, 19]. SO might partly explain the high prevalence of lean NAFLD in patients with T2DM. No clinical data to date have revealed the influence of sarcopenia alone, visceral obesity alone, or sarcopenic visceral obesity (SV) on lean NAFLD based on diabetic population. Thereby, the aim of our study was to explore the associations of sarcopenia, visceral obesity, and SV with lean NAFLD focusing on Chinese patients with T2DM.

## 2. Materials and Methods

### 2.1. Study Subjects

A total of 1,306 T2DM patients with BMI < 25 kg/m^2^ were initially enrolled from the National Metabolic Management Center (MMC) of the First Affiliated Hospital of Wenzhou Medical University, from March 2017 to March 2021. Then, subjects with missing data of the appendicular skeletal muscle mass (ASM), visceral fat area (VFA), or abdominal ultrasonography were excluded. Next, subjects with alcohol consumption > 140 g/week for men and >70 g/week for women or with other primary liver diseases, such as hepatitis and malignant or autoimmune liver diseases, were also excluded. Since the great majority of enrolled MMC subjects were hospitalized in the endocrinology department of our hospital, our main judgment criteria for other primary liver diseases were based on the patients' hospitalization diagnosis, which were according to the patients' past medical history and existing laboratory results. Finally, 1,112 subjects were enrolled in this cross-sectional study.

Written informed consents were obtained from all study participants, and the current study was approved by the Ethics Committee in Clinical Research of the First Affiliated Hospital of Wenzhou Medical University (No. KY2021-173).

### 2.2. Data Collection

Brachial systolic blood pressure (SBP) and diastolic blood pressure (DBP) were taken after sitting for at least 5 minutes. Body weight, height, waist circumference (WC), and hip circumference (HC) were measured by trained staff according to consistent ways. BMI was calculated as body weight (kg) divided by height squared (m^2^). WC was measured at the midpoint between the lowest rib and the iliac crest; HC was measured at the level of maximum extension of the hip.

Blood samples were collected after overnight fasting for at least 8 hours. Fasting blood glucose (FBG), glycated hemoglobin A1c (HbA1c), triglyceride (TG), total cholesterol (TC), high-density lipoprotein cholesterol (HDL-C), low-density lipoprotein cholesterol (LDL-C), alanine aminotransferase (ALT), aspartate aminotransferase (AST), serum creatinine (Cr), and uric acid (UA) were assayed with an automatic biochemical analyzer (Beckmann AU5800). IR was assessed according to the homeostasis model assessment [20]. The definition of dyslipidemia was TG ≥ 2.3 mmol/L, TC ≥ 6.2 mmol/L, HDL‐c < 1.0 mmol/L, LDL‐c ≥ 4.1 mmol/L, non‐HDL‐c ≥ 4.9 mmol/L, or underwent antihyperlipidemic medication currently as suggested by the 2016 Chinese Guidelines for the Management of Dyslipidemia in Adults [21].

Information on alcohol consumption, smoking status, and diabetes durations was collected by using a standardized questionnaire.

### 2.3. Assessments of Body Compositions

Body compositions markers were assessed by a dual bioelectrical impedance analyzer (BIA) (InBody 720; Biospace, Land Seoul, Korea), which is a valid tool and a good alternative comparing to dual-energy X-ray absorptiometry (DXA) [22, 23]. ASM was calculated as the sum of lean muscle mass of four limbs. Skeletal muscle mass index (SMI) was applied as weight-adjusted ASM (SMI = ASM/body weight × 100%) [24, 25]. We adopted the definition of sarcopenia as <1 standard deviation (SD) below the sex-specific average for a young reference population from the Korean datasets of KNHANES 2008–2011 since no such data were available in Chinese population, and the cut-off points for sarcopenia were 32.2% for men and 25.5% for women [26]. Visceral obesity was defined as VFA ≥ 100 cm^2^ in both gender [27–29]. All participants were divided into four groups based on above statements: nonsarcopenia/nonvisceral obesity (NN); nonsarcopenia/visceral obesity (NV); sarcopenia/nonvisceral obesity (SN), and SV.

### 2.4. Definition of Lean NAFLD

Fatty liver was determined by abdominal ultrasonography, which was performed by experienced radiologists who were blind to this clinical study. After excluding for other causes of hepatic fat accumulation, such as alcoholic hepatitis and malignant or autoimmune liver diseases, patients with fatty liver were clinically diagnosed with NAFLD [30]. Among them, subjects with BMI less than 25 kg/m^2^ were considered having lean NAFLD [3].

### 2.5. Statistical Analysis

The baseline characteristics of the study participants were compared according to the status of lean NAFLD. Continuous variables were presented as mean ± standard deviation (SD) in normal distribution and median (interquartile range) in abnormal distribution. Categorical variables were showed as numbers with percentages. Difference analyses between two groups were conducted using Student's *t*-test for continuous variables with normal distribution, Mann–Whitney *U* test for skewed variables, and chi-square test for categorical variables. Multivariable logistic regression models were applied to investigate the associations of sarcopenia, visceral obesity, and SV with lean NAFLD. Interactions between sarcopenia and metabolic risk factors on lean NAFLD were tested with *p* values for interaction. All statistical analyses were performed using SPSS version 26.0 software (IBM Corporation), and *p* values were considered statistically significant when <0.05.

## 3. Results

### 3.1. Baseline Characteristics

Totally, 369 (33.18%) lean NAFLD cases and 743 (66.82%) nonlean NAFLD cases were identified in our study (Table 1). The presence of sarcopenia and visceral obesity was significantly higher in patients with lean NAFLD in contrast with those without (sarcopenia: 40.92% vs. 18.71%, *p* < 0.001; visceral obesity: 47.43% vs. 22.88%, *p* < 0.001). Compared to patients without lean NAFLD, those with lean NAFLD were more likely to be female and had shorter diabetes durations, higher blood pressures (BPs), BMI, WC, HC, HOMA-IR, TG, TC, LDL-c, ALT, AST, UA, and lower HDL-c. There were no statistical differences between two groups on account of age, smoking status, levels of FBG, HbA1c, or Cr.

### 3.2. Associations of Sarcopenia, Visceral Obesity, and SV with Lean NAFLD

**Figure 1:**
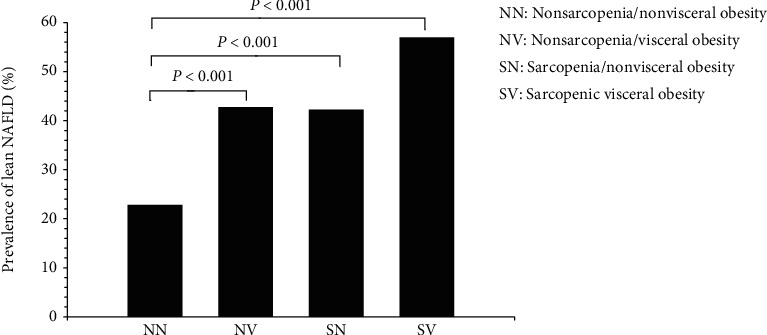
Prevalence of lean nonalcoholic fatty liver disease (NAFLD) across four phenotypes in patients with type 2 diabetes mellitus.

As presented in Figure 1, the prevalence of lean NAFLD was 22.84% in NN, 42.76% in NV, 42.27% in SN, and 56.99% in SV groups, with a higher presence of lean NAFLD in either NV, SN, or SV than NN patients (*p* < 0.001).

As shown in Table 2, compared to participants in the NN group, those in the NV and SN groups had a higher risk of having lean NAFLD (NV: OR = 1.74, 95% CI: 1.09, 2.78; SN: OR = 2.07, 95% CI: 1.23, 3.46) after adjusting for age, gender, SBP, DBP, HOMA-IR, TG, HDL-c, LDL-c, BMI, WC, diabetes durations, and smoking status. Of note, patients who suffered from both sarcopenia and visceral obesity were 3.29 times more likely to have lean NAFLD (SV: OR = 3.29, 95% CI: 2.10, 5.17) than those in the NN group after full adjustments.

### 3.3. Subgroup Analysis

Associations between sarcopenia and prevalent lean NAFLD among various metabolic risk factor subgroups are presented in Table 3. The harmful effect of sarcopenia on lean NAFLD was observed across all six subgroups, including gender, age, diabetes durations, visceral obesity, high BPs, and dyslipidemia. There were no interaction effects of metabolic risk factors on the association between sarcopenia and lean NAFLD.

## 4. Discussion

The current study suggested that SV indicated a higher risk for lean NAFLD in Chinese patients with T2DM than sarcopenia or visceral obesity alone. Moreover, our study showed that the strongly harmful effect of sarcopenia on lean NAFLD was independent of visceral obesity and other metabolic risk factors. Given that lifestyle modification is the cornerstone for the management of NAFLD [4, 31], our study proposed that we should pay more attention to the maintenance of skeletal muscle mass while reducing visceral fat in order to ameliorate lean NAFLD.

It is widely reported that NAFLD in lean individuals may be linked to the compartmentalization of fat at ectopic sites, especially at visceral one. And lean subjects with NAFLD can develop the full spectrum of metabolic comorbidities [32]. Our baseline characteristics echoed these findings well, which reflected the relative reliability of our study results. Studies have addressed that increase in skeletal muscle mass played a protective role in the development of lean NAFLD and also improved the resolution of existing lean NAFLD [33–35]. Overloaded lipid toxicity accelerated the progression from NAFLD to NASH and cirrhosis; the methods to decrease visceral fat remained critical in patients with lean NAFLD [36–38]. Therefore, it is conceivable that individuals with SV would have a significantly higher risk of lean NAFLD than either sarcopenia or visceral obesity alone. The similar pattern was also found in other studies before, which discovered that SO was much closer related to the risk of IR [39], metabolic syndrome [40], and cardiovascular diseases than sarcopenia or obesity alone [41, 42]. However, almost no study has investigated the relationship between SO and lean NAFLD. Three recent researches have demonstrated that SO was independently related to an increased risk of NAFLD in the general population [13–15], while without further distinction for lean or obese NAFLD. Only one community-based study focused on subjects with BMI < 25 kg/m^2^; in accordance with ours, it reported a 2.367-fold increased risk of lean NAFLD among subjects with vs. without SO. Total body fat mass, representing for general obesity, was used for classification of obesity in this above study [16]. Different from that, visceral obesity indicated by VFA was applied for evaluating obesity in our analysis, which was considered to be more appropriate for the diagnosis of SO than general obesity especially for lean ones [41]. Furthermore, no study to date has explored the association of SO with lean NAFLD among population with diabetes. Nevertheless, patients with diabetes tend to have higher prevalence and worse severity of lean NAFLD, and the other way round, lean NAFLD could also be an emerging risk factor for diabetes and diabetes-related complications [43]. Therefore, it is of great importance to confirm suspected lean NAFLD in patients with diabetes; our findings suggested that both low skeletal muscle mass and visceral fat mass should be taken into account.

A reduction in muscle mass and consequent decreasing physical activity levels lead to the less energy expenditure, which results in the increasing prevalence of obesity, particularly for visceral fat accumulation [44]. On the other hand, the accumulation of visceral adipose tissue stimulates the systemic inflammatory response and reactive oxygen species, both of them act significantly on the loss of muscle mass and strength [45]. Thereby, the coexistence of sarcopenia and visceral obesity could potentiate each other and maximize their adverse effects on morbidity and mortality of metabolic diseases [46]. The complicated interaction between skeletal muscle, liver, and adipose tissue, known as the muscle-liver-adipose tissue axis, plays an essential role in the pathophysiological course of NAFLD, and it may explain why SO triggers worse hepatic steatosis [47]. Firstly, IR caused by excessive adipose tissue results in the increasing free fatty acids (FFAs) and de novo lipogenesis in the liver [48]. Meanwhile, researches suggested that obesity-related IR might impede skeletal muscle growth and accelerate muscle protein degradation by impairing the mTOR pathway [49, 50]. Studies also showed that the increased lipid metabolites caused by adiposity could result in impaired insulin signaling and mitochondrial dysfunction in the muscle [19]. Since muscle tissue is the primary organ for whole-body glucose homeostasis, sarcopenia itself diminishes glucose disposal, further favoring IR, which would aggravate the evolution of NAFLD [33]. Secondly, increasing circulating inflammatory cytokines induced by obesity, such as TNF-*α*, and higher leptin could lead to the inflammation and fibrosis in the liver [51]. Researches also indicated that these inflammatory cytokines were negatively correlated with skeletal muscle mass and increased the risk of muscle degradation, which would lead to the worsening of NAFLD [11, 47]. Thirdly, increased myostatin levels are involved with decreased muscle mass, which is a risk factor in the fibrosis of NAFLD by activating the fibrogenic hepatic stellate cells. Simultaneously, myostatin, via decreasing adiponectin, damaging insulin signaling, and fat oxidation, could result in the accumulation of fat mass and thus do harm to NAFLD as well [12, 52]. To summarize, the reciprocal and intricate interactions between the skeletal muscle, liver, and adipose tissue contribute to the development of NAFLD; patients with SO are more likely to have NAFLD than those with sarcopenia or obesity alone. Restoring homeostasis in the muscle-liver-adipose tissue axis is the key to improve the natural course of NAFLD.

Our present study also indicated that sarcopenia itself was linked to lean NAFLD regardless of visceral obesity or other metabolic risk factors, which were in accordance with several previous studies [26, 33, 53], though few of them were based on patients with diabetes. Evidences also showed that regular exercise was associated with less NAFLD relevant markers only in subjects with obesity who did not suffer from sarcopenia, suggesting that a certain muscle mass might be necessary to exert benefits of exercise on the amelioration of fatty liver [26]. The specific connections between skeletal muscle mass and NAFLD were discussed below. Skeletal muscle, known as an endocrine organ, could secrete various myokines to mediate crosstalks between muscle and targeted organs [54]. For instance, irisin is involved in the peroxisome proliferator-activated receptor *α* (PPAR*α*) signaling and plays a crucial part in fatty acid beta-oxidation in hepatic cells [33]. In inflammation-prone animal models, IL-6, as one of myokines, showed a beneficial effect in the development of NAFLD by downregulating lipogenic genes and upregulating fatty acid oxidation-associated genes [55]. Skeletal muscle mass could play a straight causative role in fatty liver by secreting various myokines. Lifestyle intervention on improving lean NAFLD should also lay emphasis on muscle building more than just reducing visceral fat accumulation.

The present study had several limitations. Firstly, it is a cross-sectional study; the causality of sarcopenia and visceral obesity with lean NAFLD could not be inferred. Secondly, we defined lean NAFLD using abdominal ultrasonography instead of liver biopsy, the gold standard tool for such a diagnosis. But, strong evidences have showed that ultrasonography was a well-established and cost-effective imaging technique for the diagnosis of hepatic steatosis in clinical practice [56]. In addition, though BIA is a valid tool for assessing body composition, it is still not as accurate as CT/MRI. Thirdly, tests for excluding other etiologies of liver steatosis were not further performed in our study, which might cause certain misjudgment. Fourthly, due to the unavailable measurement of muscle function such as hand-grip power, sarcopenia was only defined by the lower skeletal muscle mass in our study. Recognition of “true” sarcopenic ones in patients with T2DM, which includes both muscle mass and muscle function, should help to better elucidate the role of sarcopenia in the development of lean NAFLD. Last, since lean subjects with NAFLD have an increased rate of PNPLA3 risk allele carriage [57], further genetic investigation should be performed to better understand the relationship between body composition and lean NAFLD.

## 5. Conclusion

In summary, both low skeletal muscle mass and high visceral adiposity were significantly associated with highly prevalent lean NAFLD in Chinese patients with T2DM, and the risk was further enlarged when sarcopenia was accompanied with visceral obesity. Moreover, we found that the adverse effect of sarcopenia on lean NAFLD was not interfered by visceral obesity or other metabolic risk factors. These findings might suggest that a healthy lifestyle modification should be insisted on for the remission of NAFLD in lean subjects, which should focus on reinforcing skeletal muscle mass while reducing visceral fat accumulation. Perspective and interventional researches are warranted to testify the benefits of such lifestyle mode.

## Figures and Tables

**Table 1:** Baseline characteristics of study participants according to the status of lean nonalcoholic fatty liver disease.

	Total	Lean NAFLD+	Lean NAFLD-	*p*
*N*	1,112	369 (33.18%)	743 (66.82%)	
Male	641 (57.64%)	197 (53.39%)	444 (59.76%)	0.043
Age (years)	53.45 ± 10.71	53.45 ± 10.91	53.44 ± 10.62	0.990
Current smoking	292 (27.16%)	88 (24.65%)	204 (28.41%)	0.192
Diabetes durations (months)	76.5 (12.0, 138.0)	64.0 (5.5, 127.5)	84.0 (18.0, 149.0)	0.002
Anthropometric indices				
SBP (mmHg)	126.7 ± 20.8	129.3 ± 19.4	125.4 ± 21.4	0.002
DBP (mmHg)	73.9 ± 11.6	76.0 ± 11.4	72.8 ± 11.6	<0.001
BMI (kg/m^2)^	22.6 (21.1, 23.8)	23.4 (22.4, 24.3)	22.0 (20.5, 23.3)	<0.001
WC (cm)	83.6 ± 7.0	86.6 ± 6.1	82.1 ± 6.9	<0.001
HC (cm)	91.0 (88.0, 94.0)	92.3 (90.0, 96.0)	90.0 (86.0, 93.0)	<0.001
SMI (%)	31.25 ± 4.04	29.82 ± 3.43	31.96 ± 4.14	<0.001
Sarcopenia	290 (26.08%)	151 (40.92%)	139 (18.71%)	<0.001
VFA (cm^2)^	88.4 ± 23.2	99.1 ± 19.3	83.1 ± 23.2	<0.001
Visceral obesity	345 (31.03%)	175 (47.43%)	170 (22.88%)	<0.001
Biochemical indices				
FBG (mmol/L)	7.8 (6.2, 9.6)	7.8 (6.5, 9.3)	8.0 (6.0, 9.7)	0.779
HbA1c (%)	9.9 (8.1, 11.9)	9.8 (8.3, 11.5)	10.0 (8.1, 12.2)	0.193
HOMA-IR	2.16 (1.32, 3.48)	2.70 (1.78, 4.14)	1.82 (1.16, 3.04)	<0.001
TG (mmol/L)	1.65 (1.02, 3.20)	2.02 (1.44, 3.66)	1.42 (0.91, 2.96)	<0.001
TC (mmol/L)	4.34 (3.13, 5.41)	4.65 (3.55, 5.64)	4.23 (2.88, 5.26)	<0.001
HDL-c (mmol/L)	1.02 (0.88, 1.20)	0.97 (0.85, 1.12)	1.05 (0.89, 1.25)	<0.001
LDL-c (mmol/L)	2.69 ± 0.88	2.83 ± 0.86	2.62 ± 0.89	<0.001
ALT (IU/L)	23.0 (16.0, 28.0)	28.0 (19.0, 30.3)	21.0 (14.0, 26.7)	<0.001
AST (IU/L)	22.0 (16.0, 25.0)	24.8 (19.0, 27.0)	21.0 (16.0, 23.8)	<0.001
Cr (*μ*mol/L)	65.0 (52.0, 97.0)	65.0 (52.0, 87.0)	65.0 (52.0, 104.8)	0.471
UA (*μ*mol/L)	300.3 (258.0, 330.5)	318.9 (275.3, 351.8)	292.8 (250.5, 319.0)	<0.001

The data were displayed as mean ± standard deviation or as median (interquartile range) for continuous variables or as numbers and percentage for categorical variables. Abbreviations: lean NAFLD: lean nonalcoholic fatty liver disease; SBP: systolic blood pressure; DBP: diastolic blood pressure; BMI: body mass index; WC: waist circumference; HC: hip circumference; SMI: skeletal muscle mass index; VFA: visceral fat area; FBG: fasting plasma glucose; HbA1c: glycated hemoglobin A1c; TG: triglyceride; TC: total cholesterol; HDL-c: high-density lipoprotein cholesterol; LDL-c: low-density lipoprotein cholesterol; ALT: alanine aminotransferase; AST: aspartate aminotransferase; Cr: serum creatinine; UA: uric acid. Definitions: visceral obesity: VFA ≥ 100 cm^2^; sarcopenia: SMI < 1 standard deviation (SD) below the sex-specific average for a young reference population (male: SMI < 32.2% and female: SMI < 25.5%).

**Table 2:** Associations between four phenotypes and prevalent lean nonalcoholic fatty liver disease in patients with type 2 diabetes mellitus.

	*N* (%)	Model 1			Model 2			Model 3		
		OR	95% CI	*p*	OR	95% CI	*p*	OR	95% CI	*p*
NN	670 (60.25%)	1.00			1.00			1.00		
NV	152 (13.67%)	3.66	2.43-5.51	<0.001	1.78	1.12-2.83	0.014	1.74	1.09-2.78	0.020
SN	97 (8.72%)	2.52	1.60-3.97	<0.001	1.93	1.17-3.17	0.010	2.07	1.23-3.46	0.006
SV	193 (17.36%)	7.07	4.80-10.43	<0.001	3.46	2.21-5.40	<0.001	3.29	2.10-5.17	<0.001

Model 1: adjusted for age and gender; model 2: model 1 + SBP, DBP, HOMA − IR, TG, HDL − c, LDL − c, BMI, and WC; model 3: model 2 + diabetes durations and smoking status. Abbreviations: NN: nonsarcopenia/nonvisceral obesity; NV: nonsarcopenia/visceral obesity; SN: sarcopenia/nonvisceral obesity; SV: sarcopenic visceral obesity; SBP: systolic blood pressure; DBP: diastolic blood pressure; TG: triglyceride; HDL-c: high-density lipoprotein cholesterol; LDL-c: low-density lipoprotein cholesterol; BMI: body mass index; WC: waist circumference.

**Table 3:** Subgroup analysis between sarcopenia and prevalent lean nonalcoholic fatty liver disease in patients with type 2 diabetes mellitus.

	Lean NAFLD (%)	Sarcopenia (%)	OR	95% CI	*p* value	*p* for interaction
All	369/1,112 (33.18%)	290/1,112 (26.08%)	2.30	1.65-3.21	<0.001	
Gender						0.630
Men	197/641 (30.73%)	205/641 (31.98%)	2.46	1.61-3.76	<0.001	
Female	172/471 (36.52%)	85/471 (18.05%)	1.86	1.04-3.33	0.035	
Age						0.528
≤65 years	323/978 (33.03%)	235/978 (24.03%)	2.21	1.54-3.19	<0.001	
>65 years	46/134 (34.33%)	55/134 (41.04%)	2.78	1.11-7.00	0.029	
Diabetes durations (median)						0.309
<76.5 months	205/556 (36.87%)	144/556 (25.90%)	1.87	1.14-3.08	0.014	
≥76.5 months	164/556 (29.50%)	146/556 (26.26%)	3.04	1.89-4.88	<0.001	
Visceral obesity						0.805
VFA < 100 cm^2^	194/767 (25.29%)	97/767 (12.65%)	2.10	1.24-3.58	0.006	
VFA ≥ 100 cm^2^	175/345 (50.72%)	193/345 (55.94%)	1.84	1.14-2.98	0.013	
High BPs						0.595
<140/90 mmHg	255/810 (31.48%)	186/810 (22.96%)	2.26	1.50-3.41	<0.001	
≥140/90 mmHg	114/300 (38.00%)	103/300 (34.33%)	2.30	1.24-4.25	0.008	
Dyslipidemia						0.076
No	43/205 (20.98%)	44/205 (21.46%)	5.58	2.22-14.01	<0.001	
Yes	323/901 (35.85%)	244/901 (27.08%)	2.12	1.47-3.04	<0.001	

Adjusted for age, gender, SBP, DBP, HOMA-IR, TG, HDL-c, LDL-c, BMI, WC, diabetes durations, and smoking status. Abbreviations: lean NAFLD: lean nonalcoholic fatty liver disease; VFA: visceral fat area; BPs: blood pressures; SBP: systolic blood pressure; DBP: diastolic blood pressure; TG: triglyceride; HDL-c: high-density lipoprotein cholesterol; LDL-c: low-density lipoprotein cholesterol; BMI: body mass index; WC: waist circumference.

## Data Availability

All data included in this study are available upon request by contact with the corresponding authors.
